# RETRACTED: Mohamad et al. Secukinumab and Black Garlic Downregulate OPG/RANK/RANKL Axis and Devitalize Myocardial Interstitial Fibrosis Induced by Sunitinib in Experimental Rats. *Life* 2023, *13*, 308

**DOI:** 10.3390/life16071165

**Published:** 2026-07-14

**Authors:** Hoda E. Mohamad, Mervat E. Asker, Mohamed A. Shaheen, Nourhan M. Baraka, Omer I. Fantoukh, Abdulaziz Alqahtani, Alaa E. Salama, Yasmin K. Mahmoud

**Affiliations:** 1 Department of Biochemistry, Faculty of Pharmacy, Zagazig University, Zagazig 44519, Egypt; mervatasker@zu.edu.eg (M.E.A.); nourhanbaraka@pharmacy.zu.edu.eg (N.M.B.); yasmin_kassem@pharmacy.zu.edu.eg (Y.K.M.); 2 Department of Histology & Cell Biology, Faculty of Medicine, Zagazig University, Zagazig 44519, Egypt; mashahin@medicine.zu.edu.eg; 3 Department of Pharmacognosy, College of Pharmacy, King Saud University, Riyadh 11451, Saudi Arabia; ofantoukh@ksu.edu.sa (O.I.F.); aalqahtani@ksu.edu.sa (A.A.); 4 Department of Cardiology, Faculty of Medicine, Zagazig University, Zagazig 44519, Egypt; asaslama@zu.edu.eg

The Journal retracts the article “Secukinumab and Black Garlic Downregulate OPG/RANK/RANKL Axis and Devitalize Myocardial Interstitial Fibrosis Induced by Sunitinib in Experimental Rats” [1], cited above.

Following publication, concerns were brought to the attention of the Editorial Office regarding methodological, statistical, and data integrity issues in this publication [1].

In accordance with the journal’s standard procedures, the Editorial Office and Editorial Board conducted an investigation that confirmed substantial concerns regarding methodological reporting and data verifiability, including incorrect primer reporting and the inability to validate ELISA measurements. Although the authors cooperated during the investigation, the explanations and supporting materials provided were not deemed sufficient by the Editorial Board to resolve the identified concerns. Consequently, the Editorial Board has lost confidence in the reliability of the findings and decided to retract this publication [1], as per MDPI’s retraction policy.

This retraction was approved by the Editor-in-Chief of the journal *Life*.

The authors have been informed of this retraction and have indicated their disagreement with this decision.
